# *PelD* is required downstream of c-di-GMP for host specialization of *Pseudomonas lurida*

**DOI:** 10.1186/s12866-025-03945-1

**Published:** 2025-04-16

**Authors:** Anna Czerwinski, Julia Löwenstrom, Sören Franzenburg, Espen Elias Groth, Nancy Obeng, Hinrich Schulenburg

**Affiliations:** 1 Department of Evolutionary Ecology and Genetics, University of Kiel, 24118 Kiel, Germany; 2 Institute of Clinical Molecular Biology, University of Kiel, 24118 Kiel, Germany; 3 Department of Pneumology, LungenClinic Grosshansdorf, 22927 Großhansdorf, Germany; 4 Airway Research Center North (ARCN), German Center for Lung Research (DZL), 22927 Großhansdorf, Germany; 5 Present Address: Roche Pharma Research and Early Development, Infectious Disease, Roche Innovation Center Basel, F. Hoffmann-La Roche, Basel, 4070 Switzerland; 6 Max Planck Institute for Evolutionary Biology, 24306 Plön, Germany

**Keywords:** *pelD*, *Pseudomonas lurida*, *Caenorhabditis elegans*, c-di-GMP, Biofilm, Symbiosis, Host-microbe interaction

## Abstract

**Background:**

The bacterial second messenger c-di-GMP is known to influence the formation of biofilms and thereby persistence of pathogenic and beneficial bacteria in hosts. A previous evolution experiment with *Pseudomonas lurida* MYb11, occasional symbiont of the nematode *Caenorhabditis elegans*, led to the emergence of host-specialized variants with elevated intracellular c-di-GMP. Thus far, the molecular underpinnings of c-di-GMP-mediated host specialization were unknown in this symbiosis. Therefore, the current study aimed at identifying candidate molecular processes by combining transcriptomic and functional genetic analyses.

**Results:**

We found that MYb11 host specialists differentially expressed genes related to attachment, motility and biofilm production, including *pelD* from the *pel* gene cluster. *pelD* deletion resulted in reduced intra-host competitive fitness, lower bacterial numbers in *C. elegans* and loss of biofilm biomass.

**Conclusion:**

Our results identify *pelD* as a previously unknown key modulator of beneficial symbiont-host associations that acts downstream of c-di-GMP.

**Supplementary Information:**

The online version contains supplementary material available at 10.1186/s12866-025-03945-1.

## Background

The biology of microbe-host associations and thus the function of a microbiome has gained increasing interest in recent years [1]. In general, microbes can benefit from the space and nutrients provided by the host while protecting it from pathogens or providing metabolites and nutrients [2–4]. The molecular underpinnings of microbe-host interactions are important to understand how microbes can associate with a host and be part of a microbiome [5]. Studying individual microbe-host interactions rather than complex microbiomes can help uncover and causally link these molecular details. A well-described example is the symbiosis between *Vibrio fischeri* and the Hawaiian bobtail squid *Euprymna scolopes* [6]. The squid recruits the bacterial symbionts from the environment by using a chitin gradient as a chemoattractant, subsequently allowing the bacteria to colonize the light organ of the bobtail squid by forming a biofilm. This symbiosis is beneficial for both the host and the microbe. *V. fischeri* provides bioluminescence, which is used by the nocturnally active squid for counterillumination, while the bacteria obtain amino acids from their host [7–9]. Another example is *Caenorhabditis elegans* and its microbiome member *Pseudomonas lurida* MYb11, which can protect its host from infection with *Bacillus thuringiensis* while colonizing the gut and influencing early reproduction in the worm with its boom-and-bust life cycle [2, 10, 11]. We are only beginning to understand how microbes associate with a particular host and the molecular requirements for a stable microbe-host association across different hosts [5, 12, 13].

The persistence of microorganisms with or in a host is one of several prerequisites for the establishment of a stable microbe-host association [14]. On a molecular level, 3’,5’-Cyclic diguanylic acid (c-di-GMP) is a bacterial second messenger involved in processes such as biofilm formation, surface attachment, virulence, motility, transition from motile to sessile organisms and cell cycle regulation [15]. It contributes to bacterial adaptation to and their persistence in a new environment [16, 17]. The role of c-di-GMP-mediated adaptation of *Pseudomonas aeruginosa* to the cystic fibrosis lung is an area of ongoing research and the adaptations described include excessive biofilm formation through alginate production, loss of twitching motility, resistance to antibiotics and reduced virulence [18, 19]. Given the importance of c-di-GMP for the transition from a mobile to a sessile lifestyle, its effects on biofilm formation and flagellar function are crucial [15, 17]. In c-di-GMP-dependent biofilm formation of *P. aeruginosa*, both the *psl* and *pel* gene clusters are important for initial and mature biofilm formation, while the *alg* operon is important for mature biofilm formation [16]. PelD is encoded by the *pel* gene cluster and has been identified in Pseudomonads as a membrane-bound c-di-GMP receptor that is involved in the production of the Pel-polysaccharides [20]. In addition, c-di-GMP can regulate the rotation of flagella, bacterial motility and surface attachment. Flagellar rotation responds to changing environments and generates regulatory feedback loops that are critical for the establishment of mature biofilms [17].

Although there is increasing evidence for the importance of c-di-GMP in the formation of symbiosis [13], the role of c-di-GMP in beneficial symbioses with hosts is less well understood than in infections. Thus far, c-di-GMP levels have been shown to play an important role in the association of *V. fischeri* with the Hawaiian squid [21], *Aeromonas veronii* with the zebrafish [22], and in plant beneficial bacteria [23]. Recently, we have shown that c-di-GMP is also a key factor in the host adaptation of *Pseudomonas lurida* strain MYb11 to *C. elegans* [24]. Using an evolution experiment, we found that evolved wrinkly MYb11 isolates were host-specialized through increased short-term persistence and in vitro biofilm formation [24]. Genome analysis of the host specialists revealed mutations in the *wspE*, *wspF* and *rph* genes, which regulate intracellular c-di-GMP levels. Elevated c-di-GMP levels were demonstrated to cause the increased persistence of MYb11 and other Pseudomonads from the *C. elegans* microbiome and environment in the host [24]. Despite the importance of c-di-GMP levels for microbe-host association, it is currently unknown which downstream targets of c-di-GMP underlie the beneficial interactions upon association with *C. elegans*.

Therefore, the aim of this study is to uncover the downstream targets of c-di-GMP in host-specialized MYb11 that enable adaptation to the native *C. elegans* strain MY316. We used comparative transcriptomics of ancestral and host-specialized MYb11 in different environments (liquid and solid media in vitro) to identify differentially expressed genes, followed by gene set enrichment analysis. Based on the results, we genetically manipulated candidate genes in a representative host-specialist background of MYb11 to determine their effects on in vitro biofilm formation and persistence in the *C. elegans* MY316 host. We could show that *pelD* is required for the increased competitive fitness of the host-specialized MYb11, the total number of bacteria in the host, and also in vitro biofilm formation. We thus provide insights into the mechanism of c-di-GMP-driven adaptation of symbiotic bacteria to host association via a functional *pel* gene cluster.

## Materials and methods

### Overall strategy

Since sequencing of bacterial transcripts during colonization of *C. elegans* is generally challenging, we decided to focus on the following two-step strategy: (i) Identification of MYb11-specific c-di-GMP downstream targets by RNA-Seq of three distinct mutants with upregulated c-di-GMP under two different media conditions (of these, the solid medium conditions are related to the environmental conditions of the previous evolution experiment; [24]), and (ii) subsequent functional genetic analysis of selected MYb11 candidate genes for their ability to affect association of the bacterium with the *C. elegans* host.

### Host and bacterial strains

The experiments were performed with the *Pseudomonas lurida* strain MYb11 and its natural host *Caenorhabditis elegans* strain MY316 [25]. To prepare for the experiments with MY316, we thawed frozen worm strains (-70 °C - -80 °C, in either glycerol or DMSO stocks) and grew worms on nematode growth medium agar (NGM [26]) inoculated with *E. coli* OP50 at an OD_600_ of 3. A standard bleaching protocol was used to collect sterile and synchronized L1 larvae, which were then raised on appropriate bacterial lawns (20 °C) to the L4 stage, as indicated in the individual sections. *P. lurida* strain MYb11 were isolated originally from MY316 [25]. The host-specialized MYb11 isolates with mutations in *wspE*, *wspF* and *rph* were obtained from an earlier evolution experiment [24]. Bacteria were cultured on tryptic soy agar (TSA; 20 °C, 72 h) and tryptic soy broth (TSB; 28 °C, 150 rpm, overnight) unless otherwise stated. A list of the bacteria used and generated for genetic manipulation in this study can be found in Supplementary Table 20, Additional file 2.

### RNA sequencing

In preparation for transcriptomic analysis of ancestral and wrinkly MYb11, RNA was isolated from liquid cultures (OD_600_ = 1.8) and single colonies (20 °C, 72 h). For the liquid environment, a starter culture was prepared in 50 ml Falcon tubes. 10 ml TSB were inoculated with 100 µl of a MYb11 overnight culture and cultivated (28 °C, 150 rpm) to OD_600_ = 1.8. 1 ml of liquid cultures were centrifuged at 6000 rpm for 5 min, and pellets or colonies were resuspended in 800 µl TRI-zol™ reagent (ThermoFisher), frozen and stored (-80 °C). RNA was isolated using the Direct-zol RNA Miniprep Kit (Zymo Research). NGS analyses were performed at the Competence Center for Genome Analysis (Kiel, Germany) using Illumina stranded total RNA library preparation and NovaSeq SP 2 × 50 bp sequencing.

### Transcriptome analyses

To determine differentially expressed genes in the host-specialized *P. lurida* MYb11 compared to the ancestral MYb11, the RNA-Seq data were analyzed using high-performance computers at Kiel University Computer Center and R (version 4.1.1) with the following workflows and programs: Sequence quality control was assessed with FastQC (Babraham Institute) and MultiQC [27]. Adapter trimming was achieved with Trimmomatic [28] in paired-end mode (version 0.39, phred33, adaptersUsed = TruSeq3-PE-2.fa, seedMismatches = 2, palindromeClipThreshold = 30, simpleClipThreshold = 10, minimumadapterlength = 2, lengthHeadcrop = 5, lengthMin = 36). Read mapping and read counting were performed with EDGE-pro set for paired-end reads [29], reference genome (NZ_CP023272.1) and files for *Pseudomonas lurida* MYb11 (Pseudomonas_lurida_MYb11_6243.csv.gz) and GO annotations (*Pseudomonas lurida* MYb11 GO Term Annotations: gene_ontology_csv.csv) were obtained from Pseuomonas.com [30], mutations for the host specialists *wspE*, *wspF* and *rph* [24] were manually entered in the genome. Low read count filtering, data normalization, differential expression analysis with a negative binomial generalized linear model followed by a quasi-likelihood F-test and FDR correction for multiple testing, and Gene Set Enrichment Analysis (permutations = 1000, minimum gene set size = 2, and FDR correction for multiple testing) were performed using the R [31] packages edgeR version 4.2.1 [32] and clusterProfiler version 4.12.6 [33]. KEGG Orthology was inferred using the KAAS online tool [34] (settings: GHOSTX, pep, all pseudomonas, BBH).

### Mutant generation

A two-step allele replacement procedure based on previously described protocols [24, 35, 36] was used to delete candidate genes in the *wspE* host specialist background. In detail, ~ 700 bp long PCR amplicons surrounding each mutation were cloned into pUISacB, allowing sucrose selection. The constructs were transformed into competent *E. coli* cells and transferred to *wspE* host specialists by tri parental conjugation with an *E. coli* helper strain containing pRK2013 [37]. Primers (Supplementary Table 21, Additional file 2) were designed using SnapGene software (www.snapgene.com) and NEBuilder v2.10.2 (New England Biolabs).

### Biofilm formation

To determine the adherent biofilm biomass we used previously described protocols [24, 38]. In detail, the bacterial cultures were adjusted to OD_600_ = 0.1 with M9 buffer and diluted 1:10 with TSB. Subsequently, 180 µl of the dilutions were transferred to a 96-well, flat-bottomed polystyrene microtiter plate. The plates were incubated for 48 h at 20 °C and 125 rpm. 200 µl of 0.01% crystal violet was added, incubated for 30 min at room temperature, washed four times with 300 µl ddH2O, 200 µl of acetic acid was added and incubated for 30 min at room temperature. The absorbance was measured at 590 nm, 550 nm and 530 nm.

### Bacterial persistence in worms

We quantified the persistence in worms from the L4 stage onwards, as described before (see Early colonization, persistence and release in worms) [24]. Bacterial lawns were prepared on NGM (125 µl, OD_600_ = 2) from ancestral and mutated MYb11 (overnight cultures: 28 °C, 150 rpm). For persistence assays, 40 synchronized worms were raised on the respective bacteria (from L1 to L4 stage, 20 °C). The worms were collected with M9 buffer containing 0.025% Triton-100 and 25 mM of the paralyzing antihelminthic agent tetramisole. Worms were washed in buffer using a custom-made filter tip wash system [39] and then suspended in 200 µl of M9 and incubated for 1 h (20 °C), after which 100 µl of supernatant was collected. After homogenization with beads (1 mm zirconia), serial dilutions of worm suspension were plated and CFUs quantified. CFU/worm was calculated as the difference in CFU between worm and supernatant samples divided by the number of worms.

### Competitive fitness in MY316 host

Competition experiments were performed as described for the persistence experiments and described before (see In vivo competition assays) [24]. Co-inoculated bacteria were adjusted to OD_600_ = 2 and mixed in equal amounts before seeding as lawns on NGM agar. A MYb11 labeled with dTomato [10] was used, which corresponds to the ancestral MYb11, as no differences were observed in short-term persistence (see results). The competitive index was calculated as the ratio of CFU/worm of the evolved or generated mutants to CFU/worm of the ancestor.

### Statistical analyses

Prior to data analysis, the assumptions of parametric tests (normality, homogeneity of variances) were checked with Shapiro-Wilk and F-tests (Supplementary Tables 14–19, Additional file 2). If these were not met, non-parametric tests were applied. FDR correction was used for multiple testing. Box plots show the median (center line), the upper/lower quartiles (box boundaries) and the 1.5-fold interquartile ranges (whiskers). All analyses and plots were performed in R/RStudio [40] using the packages ggplot2 [41], VennDiagram [42], cowplot [43], plotly [44], and dplyr [45] as well as Inkscape.

## Results

### Host specialists show enriched expression of genes related to cell adhesion, flagellar function and biofilm formation

**Fig. 1:**
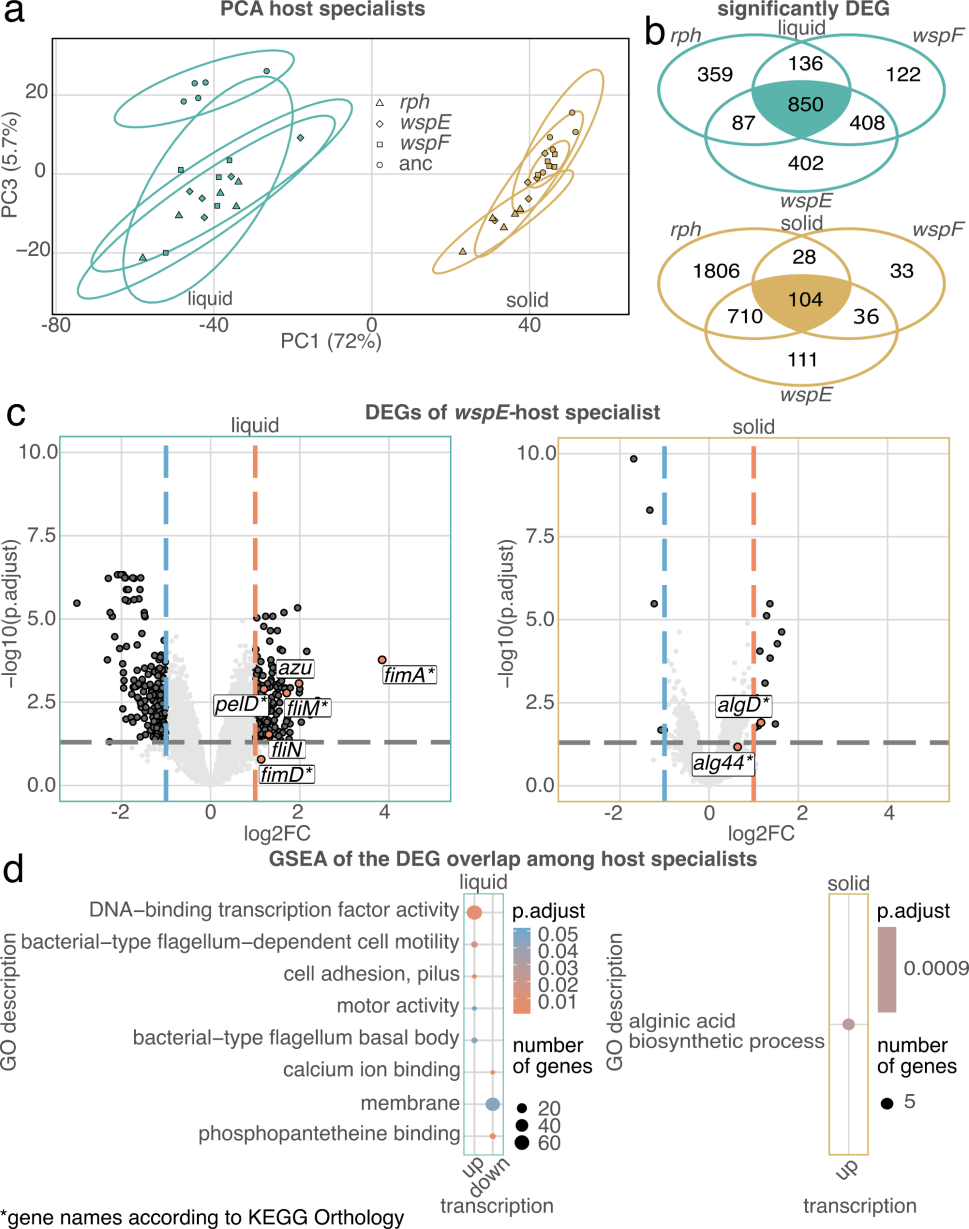
Transcriptome differences between host-specialized and ancestral *P. lurida* MYb11. **a** Principal component analysis (PCA) of log counts per million (CPM) reads of MYb11 and the evolved wrinkly *wspF*, *wspE* and *rph* mutants in liquid and solid environments (both tryptic soy based; Supplementary Table 1, Additional file 2). **b** Shared gene expression signature of the three host specialists. Venn diagrams show the significantly and differentially expressed genes of the evolved host-specialist mutants compared to the ancestral MYb11 (significantly DEG: FDR < 0.05, Supplementary Tables 8–9, Additional file 2). **c** Volcano diagram showing the DEG of the *wspE* host specialist (Supplementary Tables 2 & 5, Additional file 2). The cut-off value for the log2 change was set to -1 and 1 (blue and red dashed line), the cut-off value for the p-value to 0.05 (gray dashed line). The candidate genes selected for genetic manipulation are highlighted in red. **d** GSEA based on GO terms of the significantly DEG overlap of the host specialists (FDR < 0.05, Supplementary Tables 10–11, Additional file 2). RNA-Seq was performed with 5 independent biological replicates for each strain. Green: liquid environment, brown: solid environment. *:Indicates names annotated according to KEGG Orthology

In a previous study, we showed that c-di-GMP is a key factor for the adaptation of MYb11 to its host *C. elegans* MY316. Host adaptation was caused by mutations in the *wspE*, *wspF*, and *rph*, which all lead to elevated c-di-GMP levels [24]. However, to date, the downstream targets of c-di-GMP in this adaptation remain unresolved. As a first step to identify potential downstream targets, we performed transcriptomic analyses of ancestral- versus the three host-specialized MYb11 mutants (i.e., with mutations in the genes *wspE*, *wspF*, and *rph*) in different culture environments, including a solid environment on agar (i.e., tryptic soy agar, TSA) and a liquid medium (tryptic soy broth, TSB). A principal component analysis (PCA) of the mapped RNA-Seq counts per million (CPM) reads of ancestral and host-specialized MYb11 in the different environments showed that 72% (PC1) of the variation could be explained by the different culture environments, while 5.7% could be explained by the genomic background of the samples (PC3) (Fig. 1a, Supplementary Figs. 1–3 for PC2 and PCAs for each environmental conditions and Supplementary Table 1, Additional files 1–2). In this PCA, the samples from solid medium show a more compact distribution than those from liquid medium. Considering that the PCA uses expression data from all genes for the four strains, this result suggests that there is overall less variation among strains under solid conditions. For the subsequent differential gene expression, samples from different culture environments were analyzed separately to focus on the difference between the ancestral and host-specialized MYb11 (Fig. 1b; lists of differentially expressed genes (DEG), are given in Supplementary Tables 2–7, Additional file 2). The distribution of DEGs for the *wspE* mutant compared with the ancestor are illustrated in Fig. 1c for the two media conditions separately. A few examples of significantly DEGs are highlighted, including those analyzed further through genetic manipulation below (Fig. 1c). To characterize potential downstream functions of upregulated c-di-GMP, we then focused on the overlap of DEGs among the three mutants (850 in liquid medium, 104 DEGs on solid medium, Fig. 1b) Supplementary Tables 8–9 in Additional file 2) and subjected these gene sets to a gene set enrichment analysis (GSEA) using gene ontology (GO) terms. The results reveal eight distinct significantly enriched GO terms under liquid conditions. Of these, the GO terms cell adhesion, pilus, flagellum-dependent cell motility, flagellum basal body, and motor activity are likely important for host specialization (Fig. 1d, FDR < 0.05, Supplementary Table 10 in Additional file 2). Under solid conditions, only the GO term for alginic acid biosynthesis was significantly enriched. (Fig. 1d, FDR < 0.05, Supplementary Table 11, Additional file 2). Overall, the transcriptome analysis of the host-specialized *P. lurida* MYb11 revealed that genes related to cell adhesion, such as the fimbrial genes, flagellar function and biofilm formation, such as the *alg* and *pel* gene clusters, are potential downstream targets of c-di-GMP.

### *pelD*, but none of the other tested candidate genes contributes to MYb11 competitive fitness and biofilm formation

In a next step, we aimed to validate the role of the differentially expressed genes as downstream targets of c-di-GMP and therefore selected genes for genetic manipulation. For this, we focused on genes with (i) significant and high differential expression, and (ii) putative functions that match those identified in the GSEA (see red labelled genes in Fig. 1c; Table 1). *fimA* and *fimD* were selected as genes involved in pilus formation and surface adhesion [46]. Further, we selected *azu*, which is linked to cell copper homeostasis and type VI secretion [47]. Notably, *azu* was found to be upregulated in *P. aeruginosa* from the lungs of patients with chronic cystic fibrosis [48, 49]. The flagellar motor switch genes *fliM* and *fliN*, which are described to be involved in flagellar rotation and surface recognition [17] were chosen for the investigation of flagellar function. As genes indicative of biofilm formation and direct regulatory interaction with c-di-GMP [50], we also selected *algD*,* alg44* and *pelD*.

**Table 1:** Candidate genes for genetic manipulation in *wspE* host specialist

Name	Gene product	GO term	Gene locus_tag	Log2FC
*fimA*	type 1 fimbrial protein	cell adhesion / pilus	CLM75_RS17845	3.846
*Azu*	azurin	copper ion binding	CLM75_RS02765	1.982
*fliM*	flagellar motor switch protein FliM	motor activity	CLM75_RS19420	1.705
*fliN*	flagellar motor switch protein FliN	motor activity	CLM75_RS19415	1.306
*pelD*	sugar transporter	-	CLM75_RS01470	1.194
*algD*	nucleotide sugar dehydrogenase	alginic acid biosynthetic process	CLM75_RS05035	1.163
*fimD*	fimbrial protein	pilus assembly	CLM75_RS17855	1.132
*alg44*	alginate biosynthesis protein Alg44	cyclic-di-GMP binding	CLM75_RS05020	0.639

Legend to the table: The candidate genes have significant and high differential expression (FDR < 0.05, log2FC ≥ ± 1), and/or putative functions consistent with the functions identified in the GSEA (Fig. 1c and d, Supplementary Tables 8–11, Additional file 2). NZ_CP023272.1 was used as the reference genome for read mapping and enrichment with GO terms (Supplementary Table 12, Additional file 2); the names correspond to the KEGG Orthology (Supplementary Table 13, Additional file 2).

**Fig. 2:**
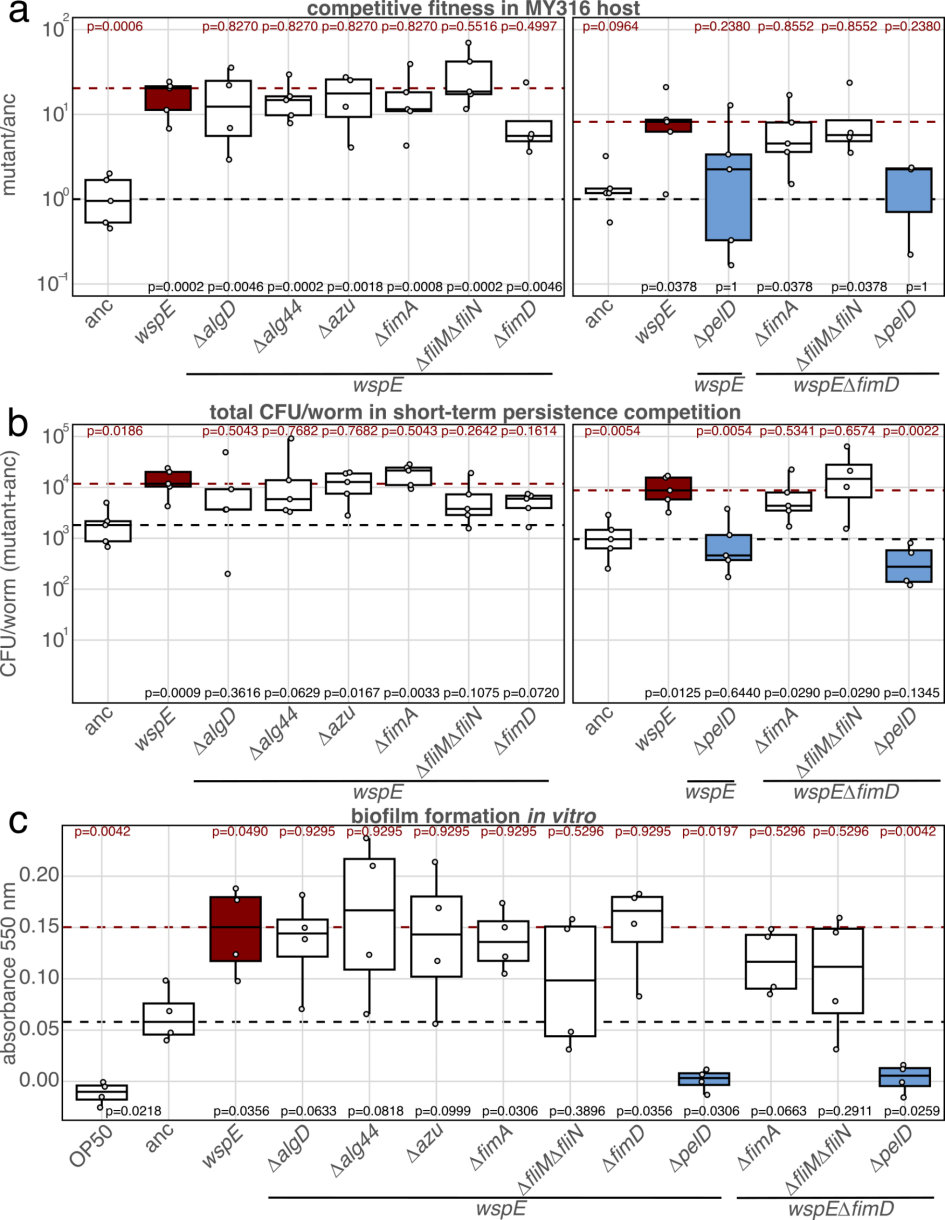
Knockout of *pelD* reduces bacterial fitness in the host and in vitro biofilm. **a** Competitive fitness of MYb11 mutants in the *C. elegans* MY316 host (CFU/worm compared to dTomato-labeled ancestor, Supplementary Table 14, Additional file 2). **b** Total CFU/per worm during short-term persistence competition in *C. elegans* MY316 (Supplementary Tables 15–16, Additional file 2). **c** Biomass of the attached biofilm, measured as crystal violet absorbance at 550 nm (Supplementary Table 19, Additional file 2). The black dashed line and the p-values at the bottom of each graph refer to comparisons with the ancestral MYb11, the red dashed line and the p-values at the top refer to the *wspE* host specialist. Ancestral MYb11, evolved *wspE-* and generated knockout mutants with *wspE* or *wspE∆fimD* background (3 < *n* < 5 replicates per strain). Statistical significance was determined using a t-test with equal variances, a Mann-Whitney U-test or a Welch’s t-test with FDR corrections for multiple testing, depending on whether the parametric assumptions were met (Supplementary Tables 14–19, Additional file 2)

After selecting candidate genes for potential c-di-GMP downstream targets, we generated gene knockouts in the *wspE* host specialist background. We focused on this specific mutant background because it showed the most robust host-specialist phenotype in various host assays [24]. We examined the competitive fitness of the generated mutants in competition with the ancestral MYb11 in the natural *C. elegans* MY316 host. Competitive fitness was performed as a short-term persistence assay and yielded data on colony-forming units (CFU) for the competing bacterial strains per worm host [24]. We found that all *∆algD*, *∆alg44*, *∆azu*, *∆fimA*, *∆fliM∆fliN* and *∆fimD* mutants maintained significantly higher competitive fitness compared to the ancestral MYb11 and thus did not lose the high competitiveness of the *wspE*-mutant host specialist (Fig. 2a left panel, Supplementary Table 14, Additional file 2).

Because the switch from a mobile to a sessile lifestyle mediated by c-di-GMP is often reflected by multiple changes in gene expression [51], we exemplarily tested whether the deletion of more than one gene was needed to reverse the host specialist phenotype. In detail, we generated *∆fimA∆fimD* to test whether we could disrupt adhesion with multiple knockouts in the same pathway, *∆fliM∆fliN∆fimD* to disrupt adhesion and different flagellar swimming modes, *∆pelD* to disrupt Pel-polysaccharides as another upregulated biofilm gene cluster, and *∆pelD∆fimD* to disrupt Pel-polysaccharide production and adhesion. Of these mutants, *∆pelD* and *∆pelD∆fimD* showed the lower ancestral competitive fitness and thus lost one of the important characteristics of the host specialist *wspE* mutant (Fig. 2a, right panel, Supplementary Table 14, Additional file 2).

In a next step, we investigated how the mutations affect the fitness of the population within the host (total number of CFU/worm as CFU/worm mutant + ancestor). The mutants *∆algD*, *Δalg44*, *∆fliM∆fliN*, *ΔfimD*, *∆pelD* and *∆pelD∆fimD* lost the significantly increased CFU/worm number in competition compared to the ancestral MYb11 (Fig. 2b). Notably, only the competitions with *∆pelD* and *∆pelD∆fimD* mutants showed a significantly reduced CFU/worm number compared to *wspE* host specialist (Fig. 2b, Supplementary Tables 15–16, Additional file 2). Similar trends, although not significant, were observed for mono-colonization of *∆pelD* and *∆pelD∆fimD* mutants compared to *wspE* host specialists (Supplementary Fig. 4, Supplementary Tables 17–18, Additional files 1–2).

In addition, we analyzed the potential of the mutants to adhere to surfaces by forming biofilms using a crystal violet microtiter plate adherence assay. We included *E. coli* OP50 as a negative control for biofilm formation [52]. The increased adherent biofilm biomass was lost in *∆algD*, *Δalg44*, *Δazu*, *∆fliM∆fliN*, *∆pelD*, *ΔfimAΔfimD*, *∆fliM∆fliN∆fimD* and *∆pelD∆fimD* mutants compared to ancestral *P. lurida* MYb11. However, a significant decrease was only observed for *∆pelD* and *∆pelD∆fimD* as compared to the ancestral MYb11 and the *wspE* host specialist (Fig. 2c, Supplementary Table 19, Additional file 2).

In summary, the *∆pelD* and *∆pelD∆fimD* mutants in the *wspE* host specialist background have lost two important features of the host-specialized MYb11, namely increased competitive fitness in the host and increased biomass of the adherent biofilm. Although other genes identified in the transcriptome analyses (Fig. 1) have already been linked to c-di-GMP-mediated shifts in Pseudomonads [17, 46–50], we were only able to causally link *pelD* to the observed adaptive traits of the host-specialized *P. lurida* MYb11 (Fig. 2).

## Discussion

In this study, we characterized possible downstream targets of c-di-GMP-mediated adaptation of host-specialized *P. lurida* MYb11. Our transcriptome analysis showed that the three different host specialist mutants varied from the ancestral MYb11 strain in gene expression for functions related to cell adhesion, flagellar function and biofilm formation. Our subsequent functional genetic analysis revealed that only the deletion of *pelD* was able to reverse the host specialist phenotype in the tested traits: competitive fitness in the host, total cell number in the host, and biofilm formation. Thus, our results emphasize the importance of *pelD* for the persistence and the biofilm phenotype of MYb11 host specialists, while other functions discovered in the transcriptome analyses may only play a minor role, at least at the level of a single gene.

PelD has been shown to post-transcriptionally regulate the production of Pel-polysaccharides in *P. aeruginosa* in a c-di-GMP-dependent manner [50, 53]. c-di-GMP binds to the degenerate GGDEF domain of the inner membrane protein PelD, leading to a conformational change and thus enabling the biosynthesis of Pel-polysaccharides [50, 53]. Functional predictions of Interpro [54] retrieved from Pseudomonas.com [30] indicate that this degenerate GGDEF domain is also part of the PelD of *P. lurida* MYb11. Furthermore, it was previously shown that *pelD* is absolutely required for the production of Pel polysaccharides in *P. aeruginosa* [55], suggesting that deletion of *pelD* in *P. lurida* MYb11 also disrupts the production of Pel polysaccharides. It follows that the higher competitive fitness of host specialists, and the enhanced biofilm formation mediated by c-di-GMP are specifically and highly dependent on a functioning *pel* gene cluster and Pel-polysaccharides. Moreover, MYb11 is a Pseudomonad that harbors the major exopolysaccharide gene clusters *alg*, *wss*, *psl* and *pel* [56], but our data suggest that none of the remaining gene clusters can compensate for the production of Pel-polysaccharides, indicating that Pel-polysaccharides are the major component of the adherent biofilm of MYb11.

This study focused on the downstream targets of c-di-GMP that are important for host association. However, the host-specialized *P. lurida* MYb11 evolved in a life cycle consisting of a host-associated and a free-living phase. The lack of an effect on the host association of other upregulated genes in the host-specialized MYb11 could have several reasons: Firstly, the lack of effect could simply be explained by genomic redundancy, i.e. genes in the tested signaling pathway, but also in the entire genome, could take over the lost function. This could be the case, for example, for genes involved in the production of fimbriae. In the MYb11 genome of *P. lurida*, other genes encoding fimbrial proteins such as *fimA* and *fimD* are present (CLM75_RS01985, CLM75_RS01970 and CLM75_RS17200, Supplementary Tables 12–13, Additional file 2). On the other hand, the upregulated genes might play a role for survival in a free-living environment and be less important inside the host. For example, genes related to alginate production have been shown to be important for the adaptation and survival of *P. fluorescens* PF0-1 in dehydrated soil [57]. In another soil bacterium, *P. putida*, the regulation of flagellar rotation and thus swimming mode has been shown to be important for motility in different liquid and solid environments [58]. In contrast, azurin is used by Pseudomonads to maintain copper-ion homeostasis in combination with TonB-dependent receptors and type VI secretion systems and therefore may play a role in nutrient acquisition in a free-living environment [47].

A detailed knowledge of how microbes adapt to the host association and persist in a host is crucial for full appreciation of the formation and functioning of host-microbiome interactions. Investigating the molecular basis of these adaptations in a single bacterial-host association will help us to identify conserved mechanisms as well as species-specific differences between microbes. In our work, we use *C. elegans* MY316 and its symbiont *P. lurida* MYb11 to study bacterial adaptation to a host. c-di-GMP and Pel-polysaccharides have been shown to be involved in host association, especially of pathogenic bacteria [59]. One example is the association of *P. aeruginosa* with cystic fibrosis lung, where Pel-polysaccharides contribute to the biofilm matrix that allows the bacteria to persist in the lung and protect it from host immune defenses and antibiotics [60]. To our knowledge, Pel-polysaccharides have not yet been shown to be involved in beneficial host-microbe associations. Thus, it is conceivable that many of the mechanisms previously reported to shape the much more intensively studied interactions between host and pathogens are actually involved in any kind of symbiotic interaction, ranging from mutualistic over commensal to pathogenic associations.

Overall, the current study extends our previous work [24] by providing new insights into the mechanisms of c-di-GMP-driven host adaptation in symbioses beyond infection and demonstrates the specific role of *pelD* that acts downstream of c-di-GMP to mediate the competitive fitness of *Pseudomonas lurida* MYb11 within its nematode host *Caenorhabditis elegans* MY316.

## Electronic supplementary material

Below is the link to the electronic supplementary material.

Supplementary Material 1

Supplementary Material 2

## Data Availability

The transcriptome sequence data is available from the NCBI GEO database (http://www.ncbi.nlm.nih.gov/geo/) under the following accession number: GSE288391. Other datasets generated and analyzed during the current study are available from our pelD_c-di-GMP_host_specialization Github repository: https://github.com/evoecogen/pelD_c-di-GMP_host_specialization.git.
