# Supplementary material for: PelD is required downstream of c-di-GMP for host specialization of Pseudomonas lurida
Source: BMC Microbiol. 2025 Apr 16;25:220. doi: 10.1186/s12866-025-03945-1 (PMC12001729; doi:10.1186/s12866-025-03945-1)
Supplement: Supplementary file 1 — Supplementary Material 1 [file 12866_2025_3945_MOESM1_ESM.docx]

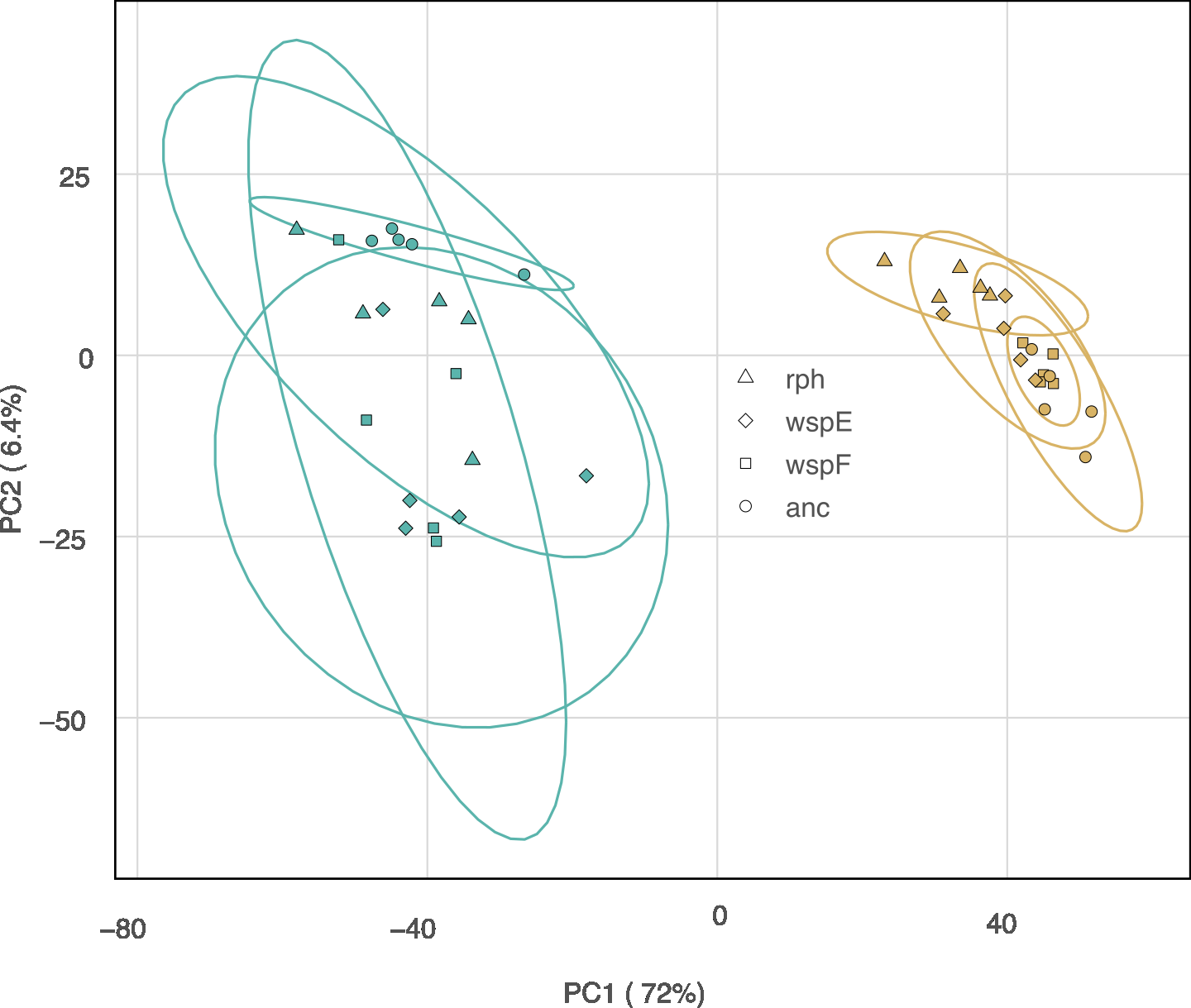


Supplementary Figure 1: PCA of ancestral and host-specialized *P. lurida* MYb11 in different environments.

PCA of log counts per million (CPM) reads of MYb11 and the evolved wrinkly *wspF*, *wspE* and *rph* mutants in liquid and solid environments with PC1 and PC2. Green: liquid environment, brown: solid environment. RNA-seq were performed with 5 replicates for each strain.


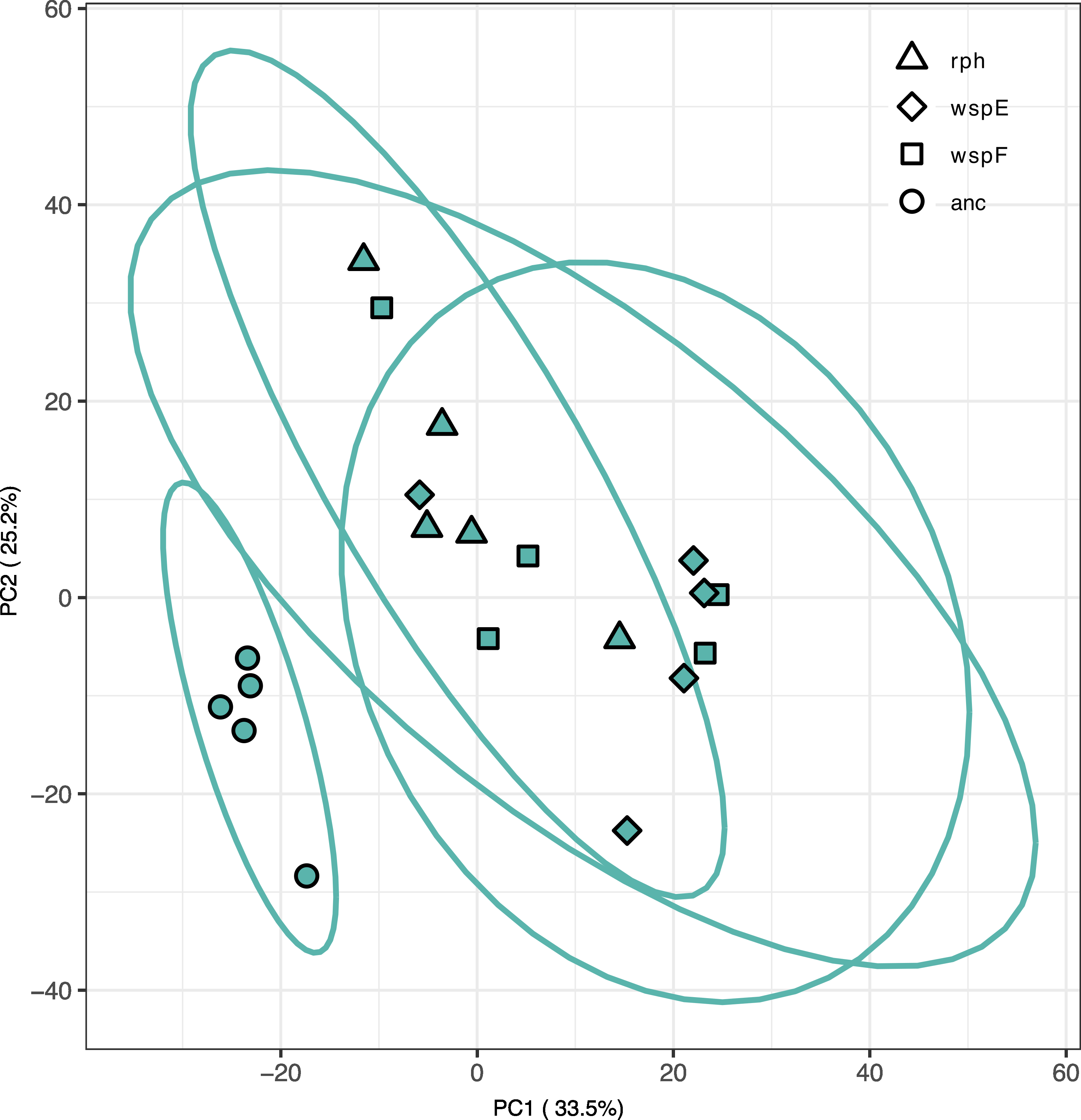


Supplementary Figure 2: PCA of ancestral and host-specialized *P. lurida* MYb11 in a liquid environment.

PCA of log counts per million (CPM) reads of MYb11 and the evolved wrinkly *wspF*, *wspE* and *rph* mutants with PC1 and PC2. RNA-seq were performed with 5 replicates for each strain.


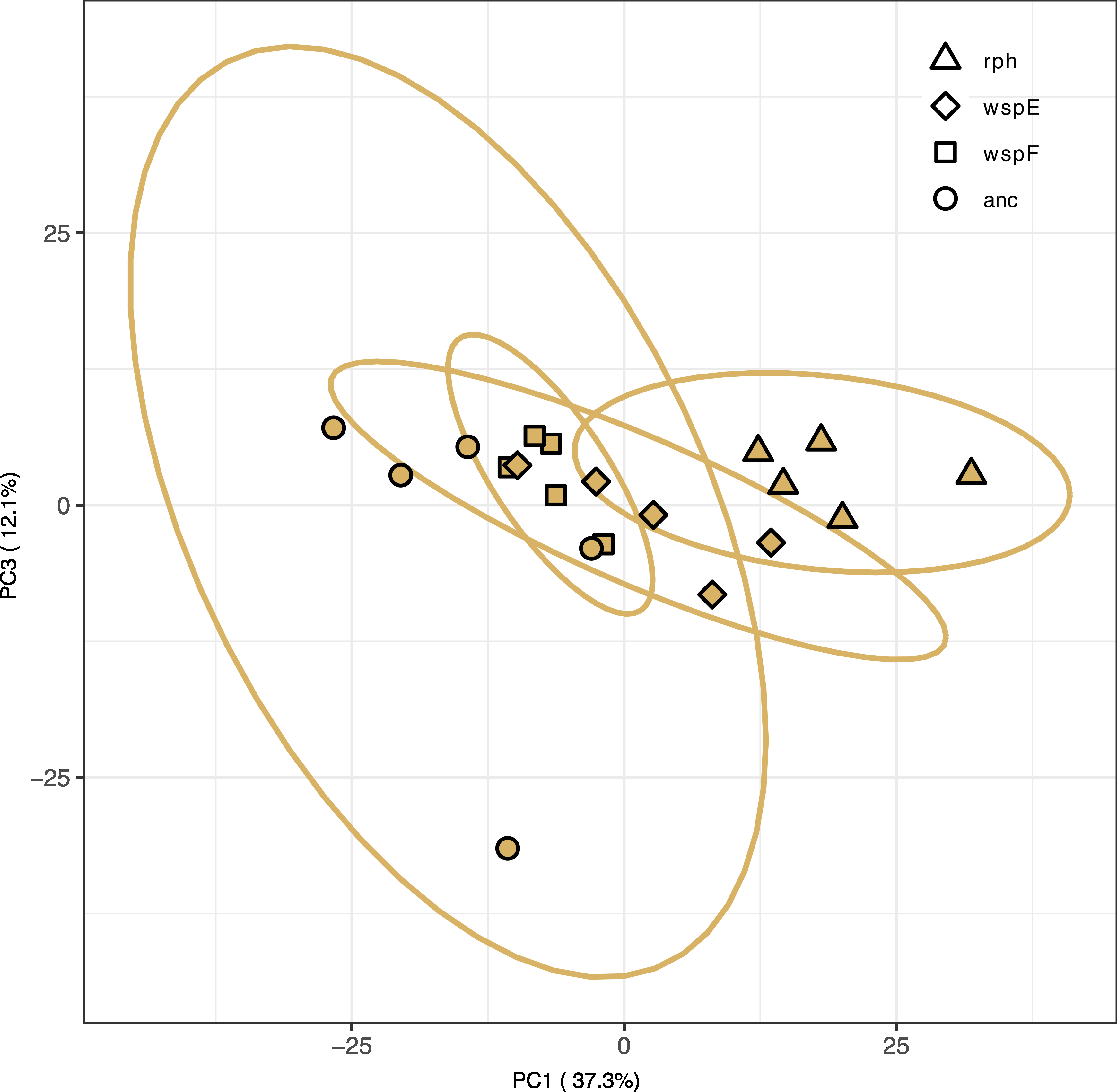


Supplementary Figure 3: PCA of ancestral and host-specialized *P. lurida* MYb11 in a solid environment.

PCA of log counts per million (CPM) reads of MYb11 and the evolved wrinkly *wspF*, *wspE* and *rph* mutants with PC1 and PC3. RNA-seq were performed with 5 replicates for each strain

**
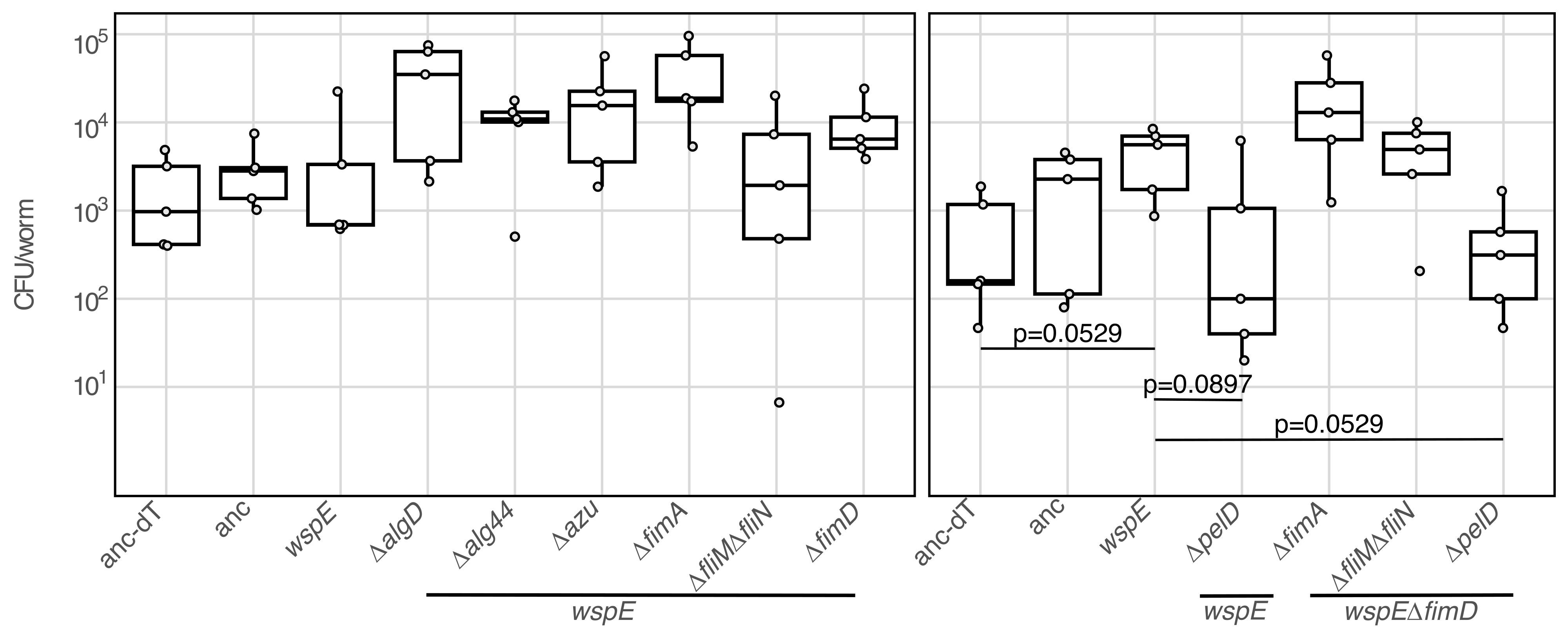
**

Supplementary Figure 4: *pelD*-knockout shows a trend towards a reduction in CFU/worm.

CFU/per worm during short-term persistence monocolonization in *C. elegans* MY316. 3 < n < 5 replicates per strain. Statistical significance was determined using an equal variances t-test, a Mann-Whitney U-test or a Welch's t-test with FDR corrections for multiple testing, depending on whether the parametric assumptions were met (Supplementary Tables 17-18, Additional file 2).
